# Beta-triggered adaptive deep brain stimulation during reaching movement in Parkinson’s disease

**DOI:** 10.1093/brain/awad233

**Published:** 2023-07-11

**Authors:** Shenghong He, Fahd Baig, Anca Merla, Flavie Torrecillos, Andrea Perera, Christoph Wiest, Jean Debarros, Moaad Benjaber, Michael G. Hart, Lucia Ricciardi, Francesca Morgante, Harutomo Hasegawa, Michael Samuel, Mark Edwards, Timothy Denison, Alek Pogosyan, Keyoumars Ashkan, Erlick Pereira, Huiling Tan

**Affiliations:** 1 MRC Brain Network Dynamics Unit, Nuffield Department of Clinical Neurosciences, University of Oxford, Oxford OX3 9DU, UK; 2 Neurosciences Research Centre, St George’s, University of London & St George’s University Hospitals NHS Foundation Trust, Institute of Molecular and Clinical Sciences, Cranmer Terrace, London SW17 0QT, UK; 3 Department of Neurosurgery, King’s College Hospital NHS Foundation Trust, London SE5 9RS, UK; 4 Department of Neurology, King’s College Hospital NHS Foundation Trust, London, SE5 9RS, UK; 5 Department of Clinical and Basic Neuroscience, Institute of Psychiatry, Psychology and Neuroscience, King’s College London, London WC2R 2LS, UK

**Keywords:** adaptive deep brain stimulation (ADBS), Parkinson’s disease (PD), reaching movement, subthalamic nucleus, beta power, gamma power

## Abstract

Subthalamic nucleus (STN) beta-triggered adaptive deep brain stimulation (ADBS) has been shown to provide clinical improvement comparable to conventional continuous DBS (CDBS) with less energy delivered to the brain and less stimulation induced side effects. However, several questions remain unanswered. First, there is a normal physiological reduction of STN beta band power just prior to and during voluntary movement. ADBS systems will therefore reduce or cease stimulation during movement in people with Parkinson’s disease and could therefore compromise motor performance compared to CDBS. Second, beta power was smoothed and estimated over a time period of 400 ms in most previous ADBS studies, but a shorter smoothing period could have the advantage of being more sensitive to changes in beta power, which could enhance motor performance. In this study, we addressed these two questions by evaluating the effectiveness of STN beta-triggered ADBS using a standard 400 ms and a shorter 200 ms smoothing window during reaching movements. Results from 13 people with Parkinson’s disease showed that reducing the smoothing window for quantifying beta did lead to shortened beta burst durations by increasing the number of beta bursts shorter than 200 ms and more frequent switching on/off of the stimulator but had no behavioural effects. Both ADBS and CDBS improved motor performance to an equivalent extent compared to no DBS. Secondary analysis revealed that there were independent effects of a decrease in beta power and an increase in gamma power in predicting faster movement speed, while a decrease in beta event related desynchronization (ERD) predicted quicker movement initiation. CDBS suppressed both beta and gamma more than ADBS, whereas beta ERD was reduced to a similar level during CDBS and ADBS compared with no DBS, which together explained the achieved similar performance improvement in reaching movements during CDBS and ADBS. In addition, ADBS significantly improved tremor compared with no DBS but was not as effective as CDBS. These results suggest that STN beta-triggered ADBS is effective in improving motor performance during reaching movements in people with Parkinson’s disease, and that shortening of the smoothing window does not result in any additional behavioural benefit. When developing ADBS systems for Parkinson’s disease, it might not be necessary to track very fast beta dynamics; combining beta, gamma, and information from motor decoding might be more beneficial with additional biomarkers needed for optimal treatment of tremor.

## Introduction

Deep brain stimulation (DBS) targeting the subthalamic nucleus (STN) has been demonstrated to be a successful treatment for patients with advanced Parkinson’s disease (PD).^1^ However, continuous DBS (CDBS) can reduce in efficacy over time and may be accompanied by stimulation related side effects such as dyskinesia, postural instability, impairment of cognition and reduced speech fluency.^2,3^

Enhanced synchronization of beta activity in the STN has been observed consistently in people with PD and is positively correlated with bradykinesia and rigidity. Conversely, improvement in bradykinesia and rigidity with medication or DBS is positively correlated with suppression of beta power.^4–9^ More recently, multiple studies have emphasized the importance of the temporal dynamics of STN beta oscillations, where the occurrence of longer beta bursts are positively correlated with motor impairment.^10–13^ Taken together, these findings suggest that STN beta activity is a biomarker for parkinsonian motor symptoms, and this has motivated the development of beta-triggered adaptive DBS (ADBS, also called closed-loop DBS) algorithms, with the aim of improving therapeutic efficacy while limiting side effects. The results of several pilot trials of ADBS with temporarily externalized DBS electrodes^8,14–20^ or chronically implanted DBS devices^21^ suggest that beta-triggered ADBS, in which the stimulation amplitude is adjusted based on real time STN beta power estimation, is at least as effective as conventional CDBS in reducing motor symptoms at rest as evaluated using the Movement Disorders Society Unified Parkinson’s Disease Rating Scale part III (MDS-UPDRS-III).

**Figure 1:**
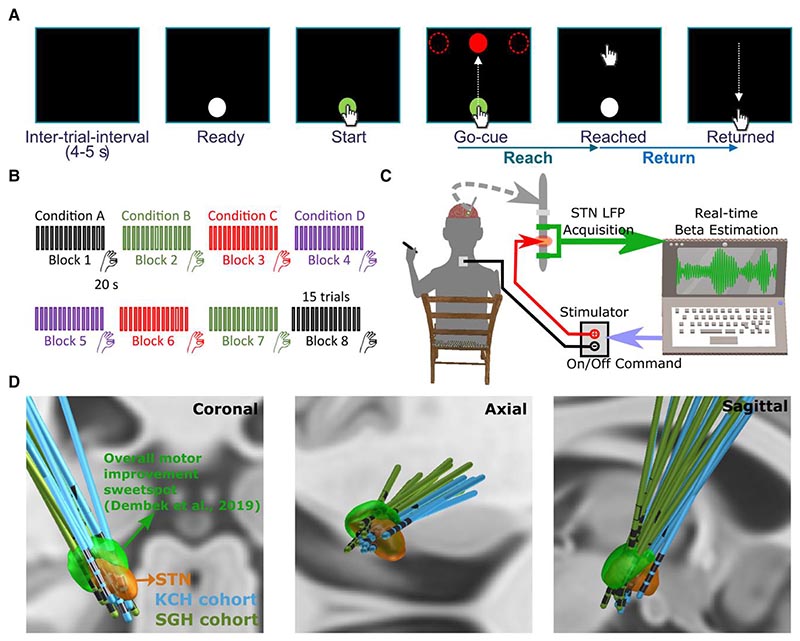
Experimental protocol. (**A**) Timeline of one individual trial of the reaching task performed on a tactile monitor with a pen. In each trial, the patient is instructed to point at the start button to initiate the trial, reach to the red target when the Go-cue is shown, and back to the start button when the target disappears, as quickly as possible. (**B**) Timeline for the whole experimental session which consists of eight counterbalanced blocks in four different stimulation conditions, with two blocks in each condition. Each block contains 15 trials of reach-return movements followed by 20 s of finger-tapping movements. (**C**) Schematic of the adaptive deep brain stimulation (ADBS) system which consists of bipolar measurement of subthalamic nucleus (STN) local field potentials (LFPs), real time estimation of beta amplitude and monopolar stimulation delivered to one of the middle contacts, while the patient is comfortably seated on a chair and performs the tasks. (**D**) 3D reconstruction in coronal (*left*), axial (*middle*) and sagittal (*right*) views of all analysed DBS leads localized in standard Montreal Neurological Institute (MNI)-152_2009b space using Lead-DBS.^28,29^ Electrodes in the left hemisphere were mirrored to the right hemisphere. The result confirmed that most of the tested electrodes clustered in a sweet spot that has been suggested to provide optimal overall motor improvement for Parkinson’s disease with DBS (shown in green).^30^ KCH = King’s College Hospital; SGH = St George’s Hospital.

However, several questions remain unanswered. First, does beta-triggered ADBS lead to worse performance in reaching movements compared with CDBS in PD patients? There is a physiological reduction of STN beta activity during voluntary movements, which is seen also in people with PD.^22–24^ In the setting of beta-triggered ADBS, this will lead to reduction or cessation of stimulation during movement. This could compromise motor performance compared with CDBS if further beta suppression during movement is helpful for maximum therapeutic benefits when patients attempt movements, which is arguably when they need it most.^25^ Second, does making the ADBS more responsive to the beta oscillation with shortened smoothing window to quantify beta amplitude lead to improvement in motor performance? The smoothing window for estimating beta is a key parameter that needs to be considered while developing ADBS, since different smoothing windows alter the dynamics of the interactions between the stimulation and the targeted oscillations. Most existing studies of beta-triggered ADBS have estimated beta amplitude in real-time using an average moving window of 400-ms duration or longer, aimed at capturing beta bursts of longer durations.^10,14–16^ Previous studies with single trial analysis of local field potentials (LFPs) recorded from striatum and motor-premotor cortex in healthy monkeys showed that brief bursts of oscillation with a duration of 50–150 ms are responsible for virtually all beta-band activity, and that most of the modulations in trial-averaged beta power primarily reflect modulations of burst density.^26^ This is consistent with results from healthy human participants showing that high-power beta events from somatosensory and frontal cortex typically lasted <150 ms and had a stereotypical non-sinusoidal waveform shape.^27^ Therefore, we hypothesized that there might be extra benefits of an ADBS algorithm capable of truncating STN beta activities into even shorter bursts, as observed in the healthy sensorimotor cortical-basal ganglia network^26,27^ via the use of a shorter smoothing time window (e.g. 200 ms). To answer these questions, we developed an experimental protocol combining a cued reaching task and a brain computer interface allowing real-time estimation of STN beta and adjustment of stimulation amplitude (Fig. 1).

We evaluated the motor performance of 13 people with PD in four different stimulation conditions: no DBS, CDBS, ADBS-400 (ADBS with beta amplitude smoothed over 400 ms) and ADBS-200 (ADBS with beta amplitude smoothed over 200 ms).

## Materials and methods

### Human subjects

From September 2021 to August 2022, 13 people with PD (six females) participated to the study after being recruited at two different centres: King’s College Hospital (KCH) and St George’s Hospital (SGH) (clinical details summarized in Supplementary Table 1). All underwent bilateral implantation of DBS electrodes targeting the motor area of the STN. The implanted DBS leads (manufacturer details in Table 1 and Supplementary material) were temporarily externalized prior to a second surgery to connect them to a neurostimulator. Lead placements were confirmed by fusion of preoperative MRI and postoperative CT scans, which were further confirmed by reconstructing the electrode trajectories and location of different contacts using the Lead-DBS MATLAB toolbox (version 2.6.0).^29^ As shown in Fig. 1D, most of the tested electrodes clustered in a sweet spot that has been suggested to provide optimal overall motor improvement for PD with DBS.^30^ One electrode appears to be at the border of the STN (P1L in Supplementary Fig. 1), so we applied volume-of-tissue activated (VTA) analysis using the stimulation parameters as used during the recording for this electrode. This analysis confirmed that stimulation applied to this electrode led to VTA that overlapped with the STN and the sweet spot for overall motor improvement. The study was approved by the local ethics committees, and all patients provided their informed written consent according to the Declaration of Helsinki. Patients participated in this study had an average age of 62.15 ± 1.58 years (mean ± SEM) and a disease duration of 10 ± 1.21 years and showed good response to dopaminergic medication with mean scores of the MDS-UPDRS-III of 37.04 ± 2.95 and 12.42 ± 1.67 for medication OFF and ON, respectively. In this study, all experiments were conducted with the patients off their dopaminergic medication for at least 6 h.

### Experimental protocol

The protocol involved two tasks: a cued reaching task performed on a Tablet Drawing Monitor (33 × 57 cm, Artist 22, XP-PEN, Japan) with a stylus pen, and a 20 s finger-tapping task. The reaching task was programmed in C# (Visual Studio 2013). As shown in Fig. 1A, each trial of the reaching task started with presentation of a white-filled circle at the bottom of the monitor indicating that the patient should bring the pen to the starting position when they were ready (Ready Cue). Once the pen was in the starting position, the circle turned green to indicate that the pen was detected. After a variable delay of 1–2 s, a red-filled circle (the Go-cue) appeared on one of the three potential target positions (top-left, top-middle or top-right of the monitor). Following this Go-cue signal, the patient was instructed to reach the target and come back to the start position as quickly as possible (Supplementary Video 1). As shown in Fig. 1B, the whole experimental session consisted of eight blocks of 15 trials, with an intertrial interval of 4–5 s (randomized). There were two blocks in each of the four tested stimulation conditions (no DBS, CDBS, ADBS-200, ADBS-400; details in next section). After the reaching movement task, and at the end of each block, the patient was asked to perform finger-tapping movements for 20 s by tapping their index fingers on their thumbs as wide and fast as possible. After changing each condition, an average interval of 67.67 ± 9.20 s (mean ± SEM) was included before starting a new block for washing out the potential stimulation effect from the previous block. In total, the recordings with each patient lasted up to 3 h for two hemispheres or 2 h for only one hemisphere. The order of the experimental blocks was pseudo-randomized and counterbalanced across patients. To achieve this, for each patient, the first four blocks included the four stimulation conditions in randomized order, and the four conditions were repeated in reverse order in the second four blocks (Fig. 1B).

### Stimulation

Stimulation was applied unilaterally to the hemisphere contralateral to the hand performing the task. A highly configurable custom-built neurostimulator certified by the University of Oxford, UK (an improved version based on what was used in previous reports^14,15^), was used to deliver constant current stimulation in monopolar mode. One of the two contacts in the middle was used as the stimulation contact, and an electrode patch attached to the back of the patient was used for reference (Fig. 1C). In cases of directional leads, the segmented contacts were used in ring mode. For those electrodes with more than four levels, only the most inferior four levels, which were supposed to locate in STN based on imaging data, were considered for stimulation/recording in this study. The stimulation had a fixed frequency of 130 Hz, a biphasic pulse width of 60 μs and an interphase gap of 20 μs. Four different stimulation conditions were considered in this study, including no DBS, continuous DBS (CDBS), adaptive DBS with the stimulator controlled by the beta amplitude estimated in real-time using a 200-ms smoothing window (ADBS-200) and adaptive DBS with a 400-ms smoothing window (ADBS-400). Before smoothing, the bipolar LFPs were filtered at the selected beta frequency band and rectified.^10,14,15^ The implementation of ADBS was the same as in previous studies,^14,15^ apart from using an advanced stimulator and adding a new condition with shorter smoothing windows (ADBS-200) to capture faster beta dynamics. To mitigate transient effects resulting in a reentrant stimulation loop during ADBS,^31^ ramping was applied at the start and end of each stimulation switching event, which forced the stimulation amplitude to linearly increase to the desired value or decrease to zero within 250 ms. In addition, a refractory time window of 50 ms was set after stimulation was switched off.

### Selecting stimulation contact and amplitude, and the beta frequency band for feedback

We followed a similar procedure used in a previous study^14^ to select the stimulation contact and amplitude, and the beta frequency band as the feedback signal. Specifically, we delivered continuous DBS to one of the middle two contacts initially at 0.5 mA. We then progressively increased the amplitude in 0.5 mA increments, until clinical benefit was seen without side effects such as paraesthesia, or until 3.5 mA was reached as the maximum amplitude. If no apparent clinical effect was observed, we repeated this procedure for the other middle contact level. Once the stimulation contact and amplitude were selected, a period of 2 min of rest recordings was performed. LFPs were recorded from two contacts neighbouring the selected stimulating contact in the differential bipolar mode. To select the individualized beta frequency band for feedback, the recorded LFPs were first notch-filtered at 50 Hz and bandpass filtered between 1 and 95 Hz using a second order zero-phase digital filter. The periodogram power spectral density (PSD) was then estimated. The feedback beta frequency band was selected as ±3 Hz around the largest beta peak (13–30 Hz). In the ADBS conditions, the threshold for triggering the stimulation was set manually for each hemisphere separately so that the DBS would be switched on for about 50% of the time when the patient was at rest (Fig. 1C) as in previous ADBS studies.^8,10,14,15,18,32^ For patients who performed the tasks with both hands, the stimulation contact and amplitude, as well as the beta frequency band and triggering threshold, were selected separately for each hemisphere. These stimulation parameters (summarized in Table 1) were kept constant for different stimulation conditions for each hemisphere. *Post hoc* comparisons confirmed that all contacts tested in this study (100%) appeared to be at least at adjacent levels of the contacts used in chronic DBS, with 66.67% of them appeared to be from the same level.

### Data recording

All recordings were carried out 3–6 days after the first surgery for DBS electrode implantation. A TMSi Porti or Saga amplifier (TMS International) was used to record bipolar LFPs from the two contacts adjacent to the stimulating contact (Fig. 1C) at a sampling rate of 2048 Hz (Cases 1–2 and 4–8; Porti amplifier) or 4096 Hz (Cases 3 and 9–13; Saga amplifier). The acceleration of the patient moving their hand was measured using a triaxial accelerometer taped to the back of the index finger and simultaneously recorded with the same amplifier at the same sampling frequency as the LFP signals. The precise timing of all cue signals of the reaching task (Start, Go, Reached and Back; Fig. 1A) and the finger tapping task (Start/Stop) were captured using a photodiode taped to the monitor and recorded with the same amplifier. Furthermore, the instantaneous stimulation amplitude applied during the real-time experiment was also simultaneously recorded by a custom-developed C program. The ground electrode was placed on the resting forearm of the patient. The *x* and *y* coordinates of the stylus on the monitor and the corresponding timestamps were recorded automatically at an irregular sampling rate of 84.3062 ± 3.3060 Hz (mean ± SEM) by a custom-developed C# program (irregularity of sampling was due to the imprecision of the timer in C#). In addition, videos of the finger-tapping movements were recorded using a smartphone (iPhone 6s; Apple Inc., US) for further blinded assessment. Among the 13 patients, seven (Cases 2–3, 7 and 10–13) performed the task with both hands separately, resulting in 20 hemispheres in total. However, the left hemisphere for Case 2 was excluded due to strong stimulation artefact contaminating the estimated beta in all stimulation conditions, probably due to the high amplitude of stimulation (3.5 mA) and/or high electrode impedance. Case 5 was excluded due to obvious stimulation induced dyskinesia even at low stimulation amplitude (1.5 mA). The data from the remaining 12 patients (18 hemispheres) were analysed. Owing to limited time for conducting the experiment, Case 10 did not perform the task in the ADBS-200 condition.

### Kinematic data analysis

#### Reaching movements

The trajectories of the reaching movements were re-constructed for each trial, based on the recorded *xy* coordinates and timestamps as shown in Figs 3A and 4A. The mean velocities of the reach and return movements were calculated separately for each trial by dividing the accumulated distances against the durations of the movements. Instantaneous velocity was quantified using two adjacent coordinates and their timestamps. In addition, the reaction time was defined as the time from the Go-cue to the first timestamp when the pen moved out of the target button.

#### Finger-tapping

For each finger-tapping movement, we quantified the root-meansquare acceleration based on the recorded three-axes accelerometer signals and acquired the mean blinded ratings from two experienced movement disorder specialists (authors F.B. and A.M.) based on the recorded video, as overall evaluations of the tapping performance (detailed in the Supplementary material).^33,34^

Resting tremor was quantified based on accelerometer measurements (detailed in the Supplementary material).

### Stimulation and local field potential data analysis

During ADBS-200 and ADBS-400, the average percentage of time when the stimulation was on and stimulation switching rate (number of stimulation events per second) were quantified based on the recorded stimulation amplitude.

The effects of the two different ADBS algorithms on the dynamics of the beta oscillations were also analysed. The bipolar LFPs recorded from the feedback channel for each task were processed offline in the same way as used for real-time beta estimation, with the only difference that a 200-ms smoothing window was used for all conditions, so that we could compare dynamics of beta oscillations across stimulation conditions. Then, the 75th percentile of the beta amplitude with the patient at rest and stimulation off was used to define beta bursts. Next, average burst duration and burst rate (events per second) were quantified as described before.^10,35^ To investigate the movement related modulation in the STN, LFPs were first epoched starting 5 s before the Go-cue to 2 s after the pen returned to the start button. Then, the signals were preprocessed, decomposed into time-frequency domain using continuous wavelet transformation and the relative changes in different frequency bands were quantified (more details in the Supplementary material). To investigate the associations between STN beta/gamma power and motor performance, for each individual trial, we first quantified beta power at different time windows, including average beta power in the 1 to 0.5 s window (W1 in Fig. 5D) before movement initiation (*β*_*w*1_) as baseline, average beta power in the 0.2 s window (W2 in Fig. 5D) around movement initiation (*β*_*w*2_), where beta was minimal, and beta event-related desynchronization (ERD) as the difference between *β*_*w*1_ and *β*_*w*__2_. Then, we used each of these beta power windows, together with stimulation condition index, as independent variables to predict the reaction time of the reaching movements in separate generalized linear mixed effect (GLME) models. In addition, the average beta power during movement (from reach/return movement onset to target reached), average gamma power during movement, stimulation condition index and reach or return index were also used as independent variables in GLME models to predict mean velocities of the reaching movements.

### Statistical analysis

Statistical analyses were conducted using custom-written scripts in MATLAB R2021-b (The MathWorks Inc, Nantucket, MA).

For those metrics quantified on a per condition basis (including stimulation switching rate, average percentage of time when the stimulation was on, average burst duration and burst rate), paired *t*-tests were used to evaluate the effect of the stimulation condition. The normal distribution assumption was tested using an Anderson–Darling test. Multiple comparisons applied to different measurements were corrected using Bonferroni correction. For each comparison, the number of cases, *t*-values and pre-corrected *P*-values were reported.

For those metrics quantified on an individual trial/block basis (including reaction time, mean velocity, rest tremor power, root-mean-square acceleration and blinded video rating), GLME modelling was used to investigate the effect of different stimulation conditions.^36^ Owing to the naturally skewed characteristic of reaction time, normal distribution with log link function was used in the models using reaction time as the dependent variable. Otherwise, normal distribution with identity link function was used. We also used GLME to further investigate the effects of STN beta/gamma power on performance of the reaching movement measured by reaction time and mean velocity on a trial-by-trial basis. In each model, the slope(s) between the predictor(s) and the dependent variable were set to be fixed across all hemispheres while a random intercept was set to vary by hemisphere. Multiple comparisons applied to different measurements were corrected using Bonferroni correction. For each GLME model, the parameters were estimated based on maximum-likelihood using Laplace approximation, the Akaike information criterion (AIC), estimated value with standard error of the coefficient (*k* ± SE), pre-corrected *P*-value and proportion of variability in the response explained by the fitted model (*R*^2^) were reported.

A chi-squared reference distribution based likelihood ratio test was conducted for the comparison of two fitted GLME models, and the likelihood ratio test statistic (LRStat), difference in degrees of freedom between two models (deltaDF) and *P*-value for the likelihood ratio test were reported for each pair of models comparison. The modelling is further detailed below together with the results.

To compare the group averaged beta/gamma power at different time points relative to movement, a non-parametric cluster-based permutation procedure (repeated 1000 times) was applied, and multiple comparisons were controlled.^37^

## Results

### No difference in motor performance between ADBS-200 and ADBS-400

**Figure 2:**
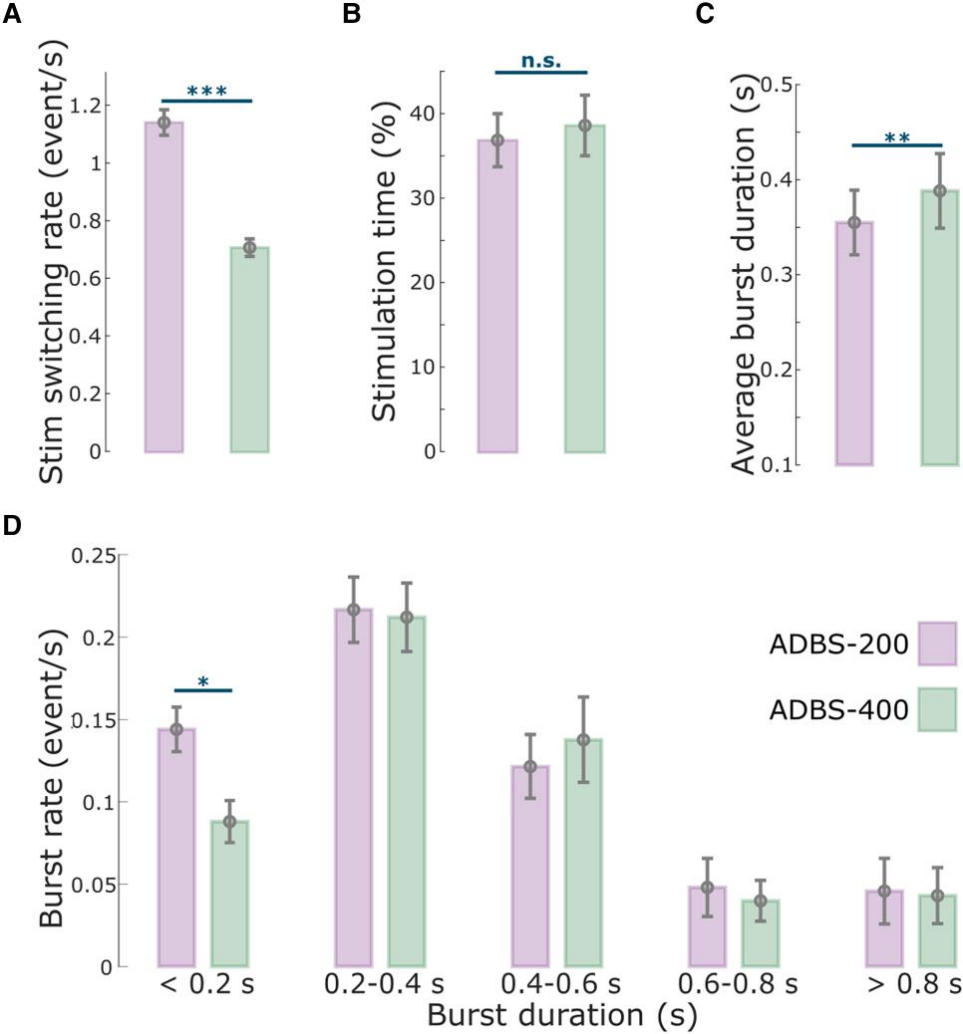
Comparison of the stimulation events and beta bursts between ADBS-200 and ADBS-400 conditions. (**A**) Averaged stimulation switching rate, (**B**) percentage of time when the stimulation was on, (**C**) averaged duration of beta bursts and (**D**) averaged rate of beta bursts with different durations in ADBS-200 (purple) and ADBS-400 (green) conditions. The error bar plots show the mean and SEM across all tested hemispheres under different conditions. *P < 0.05; **P < 0.01; ***P < 0.001; *P*-values were quantified based on paired *t*-test on individual hemisphere basis (*n* = 16) and corrected for multiple comparisons using Bonferroni correction. ADBS = adaptive deep brain stimulation; n.s. = not significant; SEM = standard error of the mean.

As expected, the stimulation was overall switched on and off more frequently during ADBS-200 compared with ADBS-400 [*t*(15) = 16.5321, *P* = 4.8823 × 10^−11^, paired *t*-test; Fig. 2A], with a trend towards a higher average percentage of stimulation on time during ADBS-400 but was not statistically significant [*t*(15) = –2.1327, *P* = 0.050, paired *t*-test; Fig. 2B].

**Figure 3:**
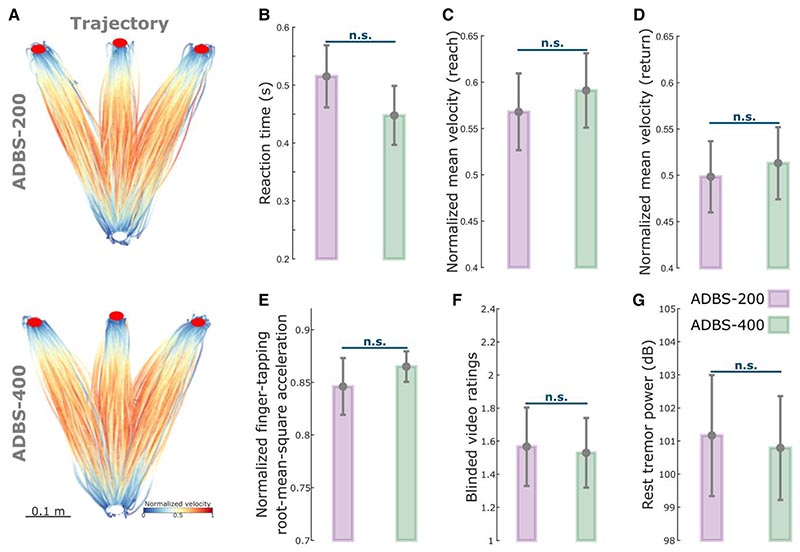
No significant difference in motor performance between ADBS-200 and ADBS-400 conditions. (**A**) Movement trajectories colour-coded by the instantaneous velocities of the reaching movement under ADBS-200 (*top*) and ADBS-400 (*bottom*) conditions. The velocities were normalized to the individual maximum of each patient. White and red filled circles at the *bottom* and *top* indicate the start and target buttons, respectively. (**B**) Reaction time during the reaching movement under different stimulation conditions. (**C** and **D**) Mean velocities during the reaching movement under different stimulation conditions, while **C** and **D** represent the reach and return periods, respectively. (**E**) Normalized root-mean-square acceleration and (**F**) blinded video ratings recorded by two experts during finger-tapping movement under different stimulation conditions. (**G**) Average power in tremor frequency band during rest under different stimulation conditions. The error bar plots show the mean and SEM across all tested hemispheres under different conditions. *P*-values were quantified using generalized linear mixed effect modelling on an individual trial (**B**, **C**, **D** and **G**) or block (**E** and **F**) basis. ADBS = adaptive deep brain stimulation; n.s. = not significant.

Despite the clear difference in the stimulator switching rate between ADBS-200 and ADBS-400 as expected from a shorter beta smoothing window, there was no significant difference in motor performance of the reaching task, including reaction time (*k* = −0.0225 ± 0.0181, *P* = 0.2155; Fig. 3B) or mean velocity (Reach: *k* = 0.0065 ± 0.0062, *P* = 0.2977; Return: *k* = 0.0025 ± 0.0053, *P* = 0.6330; Fig. 3C and D). Similarly, the two ADBS conditions led to similar performance in the finger-tapping task as evaluated by the root-mean-square acceleration *(k* = 0.0189 ± 0.0310, *P* = 0.5416; Fig. 3E) and blinded video ratings (*k* = 0.0047 ± 0.1265, *P* = 0.9703; Fig. 3F). There was no difference in resting tremor either (*k* = −0.1683 ± 0.2336, *P* = 0.4714; Fig. 3G) between the two ADBS conditions.

Then we compared how these two ADBS conditions modulated the temporal dynamics of beta oscillations. The average beta burst duration was shorter during ADBS-200 compared with ADBS-400 [*t*(15) = −2.9817, *P* = 0.0093, paired *t*-test; Fig. 2C]. This was mainly due to more bursts with shorter durations during ADBS-200 [<0.2 s, *t*(15) = 3.0478, *P* = 0.0081, paired *t*-test; Fig. 2D], and there was no significant difference for bursts with longer durations (>0.2 s) between these two conditions. Please note that here beta bursts were re-quantified offline using the same method based on the recorded bipolar LFPs using a 200-ms smoothing window for both ADBS conditions. Even though the fast ADBS-200 cut the beta burst even shorter than the ADBS-400, this faster algorithm did not further improve motor performance. These results confirm the findings of previous studies showing that only long beta bursts are pathological.

### ADBS and CDBS equally improved motor performance compared with no DBS but not resting tremor

**Figure 4:**
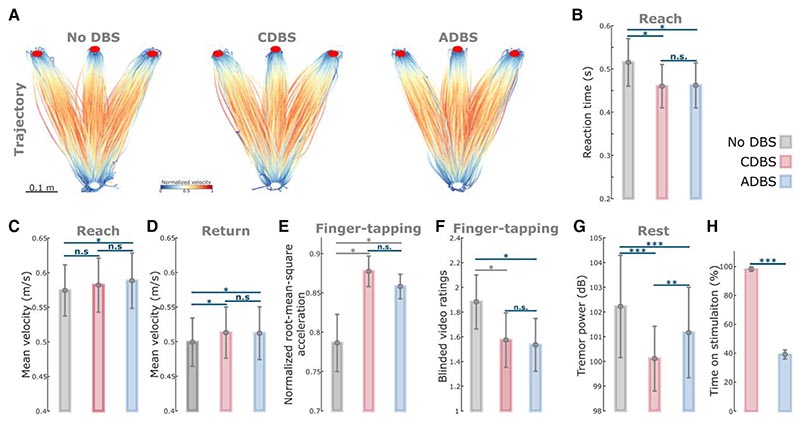
ADBS and CDBS equally improved motor performance compared with no DBS, but resting tremor was better suppressed during CDBS. (**A**) Movement trajectories are colour-coded by the normalized instantaneous velocities of the reaching movements with no DBS (*left*), CDBS (*middle*) and ADBS (*right*). White and red filled circles at the *bottom* and *top* indicate the start and target buttons, respectively. (**B**) Reaction time during the reaching movement under different stimulation conditions. (**C** and **D**) Mean velocities during the reaching movement under different stimulation conditions, while **C** and **D** represent the reach and return periods, respectively. (**E** and **F**) Normalized root-mean-square acceleration (**E**) and blinded video ratings (**F**) during finger-tapping movement in different stimulation conditions. (**G**) Average power in tremor frequency band during rest in different stimulation conditions. (**H**) Time on stimulation in CDBS and ADBS conditions. The error bar plots show the mean and SEM across all tested hemispheres; *P < 0.05, **P < 0.01; ***P < 0.001. *P*-values were quantified using generalized linear mixed effect modelling on an individual trial (**B**–**D**, and **G**) or block (**E** and **F**) basis or using paired *t*-test on an individual hemisphere basis (**H**) and corrected for multiple comparisons using Bonferroni correction. Grey asterisk indicates nominally/marginally significant, which did not survive Bonferroni correction. ADBS = adaptive deep brain stimulation; CDBS = continuous deep brain stimulation; DBS = deep brain stimulation; n.s. = not significant.

Since we did not see any behavioural difference between ADBS-200 and ADBS-400, we combined these two conditions into one ADBS condition and compared them against CDBS and no DBS for further analysis. Compared with no DBS, both CDBS and ADBS significantly improved motor performance of the cued reaching movements with reduced reaction time (CDBS versus no DBS: *k* = −0.0557 ± 0.0217, *P* = 0.0103; ADBS versus no DBS: *k* = −0.0253 ± 0.0094, *P* = 0.0072; Fig. 4B) and increased mean velocity (CDBS versus no DBS: *k* = 0.0144 ± 0.0058, *P* = 0.0139; ADBS versus no DBS: *k* = 0.0128 ± 0.0045, *P* = 0.0041; Fig. 4D) during backward movements. The effects on the mean velocity during reaching movements were smaller and only significant in ADBS (*k* = 0.0072 ± 0.0028, *P* = 0.0106; Fig. 4C) but not in CDBS (*k* = 0.0076 ± 0.0072, *P* = 0.291; Fig. 4C) conditions. Both CDBS and ADBS improved the finger-tapping movements with increased root-mean-square acceleration (CDBS versus no DBS: *k* = 0.0875 ± 0.0372, *P* = 0.0214; ADBS versus no DBS: *k* = 0.0339 ± 0.0149, *P* = 0.0253; Fig. 4E) and reduced blinded bradykinesia ratings (CDBS versus no DBS: *k* = –0.3088 ± 0.1345, *P* = 0.0249; ADBS versus no DBS: *k* = –0.1738 ± 0.0593, *P* = 0.0042; Fig. 4F and Supplementary Video 2), although some of them were only nominally/marginally significant and did not survive Bonferroni correction for multiple comparisons. When comparing between CDBS and ADBS conditions, no significant behavioural difference was found in any of the evaluated metrics (Fig. 4B–F) for the reaching or finger-tapping movements, suggesting that ADBS improved motor performance to a similar extent as CDBS. However, there was more resting tremor during ADBS compared with CDBS (*k* = 0.7605 ± 0.2179, *P* = 0.0005; Fig. 4G), even though tremor was significantly reduced in both DBS conditions compared with no DBS (CDBS versus no DBS: *k* = –2.152 ± 0.3265, *P* = 6.8335 × 10^–11^; ADBS versus no DBS: *k* = –0.5726 ± 0.1256, *P* = 5.5933 × 10^–6^; Fig. 4G). The mean duration on stimulation was only 39.39 ± 3.14% of time during ADBS, which was significantly less than CDBS where the stimulation was continuously on [*t*(17) = 18.1342, *P* = 1.4736 × 10^–12^, paired *t*-test; Fig. 4H].

### Stimulation probability during ADBS followed a similar pattern as movement-related beta modulation

**Figure 5:**
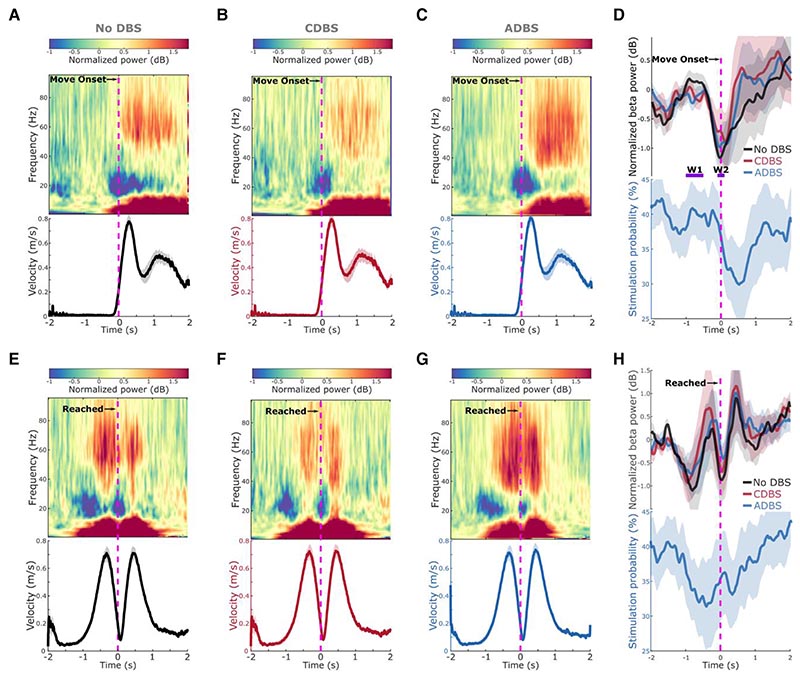
Modulation of beta/gamma power and stimulation probability during reaching movement. (**A**–**C**) Group averaged time-frequency power-spectra of the targeted STN LFPs aligned to movement onset during reaching movement under (**A**) no DBS, (**B**) CDBS and (**C**) ADBS conditions. The power spectra were normalized against a 1-s pre-Go cue resting period in each individual trial. Beta was suppressed around movement initiation and gamma was increased during movement. The *bottom* panel in each subplot indicates the group averaged velocity during the reaching movement. (**D**) Group averaged beta power in different conditions (*top*) and stimulation probability during ADBS (*bottom*) aligned to movement onset during reaching movement. Different colours indicate different conditions. Solid line and shade indicate the mean and SEM of the velocity, beta power or stimulation probability averaged across all hemispheres, respectively. W1 and W2 indicate two time-windows where the average beta power was used for predicting reaction time in Table 2. (**E**–**H**) The same as **A**–**D** but aligned to the time when the target was reached. ADBS = adaptive deep brain stimulation; CDBS = continuous deep brain stimulation; DBS = deep brain stimulation; SEM = standard error of the mean.

During all DBS conditions, a clear ERD in the beta frequency band (13–30 Hz) was observed around onset of the reaching movement (Fig. 5A–C), as well as around the time when the target was reached, before the initiation of return movements (Fig. 5E–G). In fact, the beta power reached its minimum around both reach and return movement initiations, then resynchronized to or above baseline level at the end of the movements (Fig. 5D and H). During ADBS, the averaged stimulation probability followed a similar pattern as the modulation of beta but with a constant shift in time that was caused by real-time filtering and smoothing (Fig. 5D and H). In general, the stimulation probability dropped from around 40% before the movement to 32.55 ± 4.80% in the 1-s time window after the initiation of the reaching movement in this paradigm.

### Reaction time and mean velocity during reaching movement were predicted by STN beta and gamma power

The spectrograms averaged across trials and time locked to the movement initiation also revealed clear gamma power increase during the execution of reaching movements (Fig. 5A–C and E–G). Here we further explored the potential associations between beta/gamma oscillations and motor performance, as well as the effect of different DBS protocols. Here CDBS and ADBS were combined since there was no behavioural difference between them. As shown in Table 2, the GLME modelling results suggested that although there was a positive estimation effect for *β*_*w*1_ (baseline beta) and a negative estimation effect for *β*_*w*2_ (beta around movement initiation) in predicting reaction time, neither of the effects was significant. However, there was a significant positive estimation effect on beta ERD (*k* = 0.0301 ± 0.0150, *P* = 0.0453) in predicting reaction time, together with a significant negative estimation effect on stimulation condition (*k* = –0.0742 ± 0.0363, *P* = 0.0409), suggesting stimulation and smaller beta ERDs independently predicted shorter reaction times. Non-significant interaction between stimulation condition and beta ERD suggests that the association between beta ERD and reaction time was not altered by different stimulation conditions. In addition, likelihood ratio test revealed that the GLME model using beta ERD significantly outperformed the model using *β*_*w*1_ (LRStat: 6.748; *P* < 0.001, chi-squared test) or *β*_*w*2_ (LRStat: 1.8418; *P* < 0.001, chi-squared test) in predicting reaction time.

While predicting movement velocity, GLME modelling (Model 6 in Table 2) revealed significant negative effect of beta power (*k* = –0.0042 ± 0.0008, *P* = 6.7338 × 10^–7^) and positive effect of gamma power (*k* = 0.0049 ± 0.0009, *P* = 7.7075 × 10^–9^), suggesting less beta and more gamma during movement together predicted bigger velocities. Apart from this, the modelling also revealed that the mean velocities were bigger during DBS compared with no DBS conditions (*k* = 0.0142 ± 0.0039, *P* = 0.0003), and during reach movements compared with return movements (*k* = –0.0689 ± 0.0034, *P* < 0.001), which were consistent with the results shown in Fig. 4. The GLME model combining both beta and gamma performed significantly better than the model only considered beta (LRStat: 33.35; *P* = 7.6987 × 10^−9^, chi-squared test) or gamma (LRStat: 24.685; *P* = 6.7514 × 10^−7^, chi-squared test) in predicting mean velocities, further confirming that beta and gamma simultaneously associated with the mean velocity during the reaching movement.

### DBS suppressed both STN beta and gamma, with a stronger suppression during CDBS compared with ADBS

**Figure 6:**
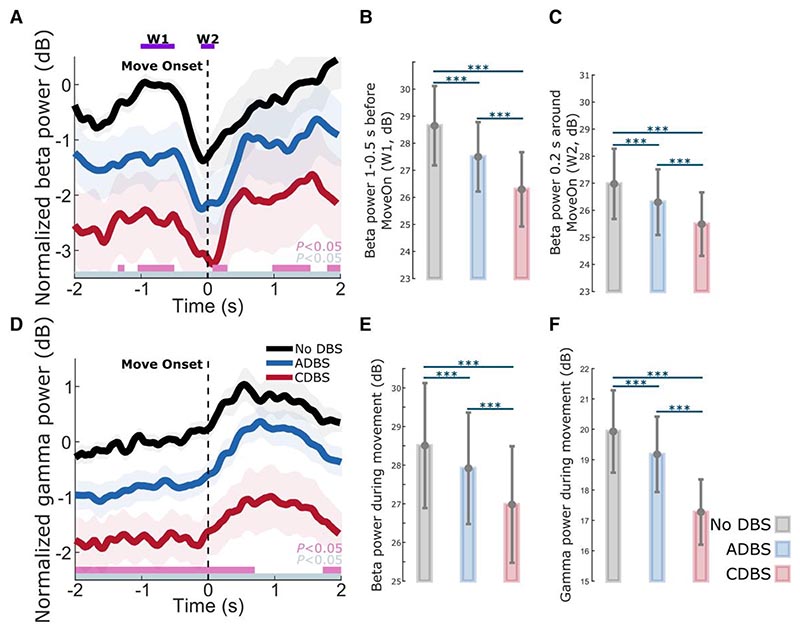
Beta and gamma power were both suppressed during DBS compared with no DBS, and the suppression was stronger during CDBS compared with ADBS. (**A**) Group averaged beta power aligned to movement onset during reaching movement under different conditions. The power was normalized against the average beta power during the 1-s pre-Go cue resting period under the no DBS condition. Solid line and shade indicate the mean and SEM of the beta power, respectively. Grey and pink bars on the *bottom* indicate the significant difference between no DBS and CDBS, and between no DBS and ADBS based on a cluster-based permutation procedure, respectively. (**B** and **C**) Averaged beta power without baseline normalization in (**B**) a baseline time window (W1, 1-0.5 s pre-Onset) and (**C**) a 0.2-s time window around movement initiation (W2) under different conditions. (**D**) The same as **A** but for gamma power. (**E** and **F**) Averaged (**E**) beta and (**F**) gamma power without baseline normalization during movement under different conditions. The error bar plots show the mean and SEM across all tested hemispheres under different conditions. *P < 0.05, **P < 0.01, ***P < 0.001. *P*-values were quantified using generalized linear mixed effect modelling on an individual trial basis and corrected for multiple comparisons using Bonferroni correction. ADBS = adaptive deep brain stimulation; CDBS = continuous deep brain stimulation; DBS = deep brain stimulation; n.s. = not significant; SEM = standard error of the mean.

As shown in Fig. 6A and D, on top of the movement related modulation, STN beta and gamma power were overall suppressed by DBS, which has been reported in previous studies,^38^ and the suppression was stronger during CDBS compared with ADBS conditions. Specifically, compared with no DBS, the suppression of beta and gamma during CDBS was significant along the whole time course, while the suppression of beta and gamma during ADBS was only significant at certain time windows. We then compared the averaged beta power in the different time windows used in Table 2 among different stimulation conditions. The results further confirmed that both ADBS (*β*_*w*1_: *k* = −0.5508 ± 0.0696, *P* = 4.8748 × 10^−15^; *β*_*w*2_: *k* = −0.3061 ± 0.0839, *P* = 0.0003; Fig. 6B and C) and CDBS (*β*_*w*1_: *k* = −2.3452 ± 0.1816, *P* = 1.5153 × 10^−35^; *β*_*w*2_: *k* = −1.4809 ± 0.1961, *P* = 9.157 × 10^−14^; Fig. 6B and C) significantly suppressed beta, and the suppression of beta was stronger during CDBS compared with ADBS (*β*_*w*1_: *k* = −1.1832 ± 0.1135, *P* = 1.2901 × 10^−24^; *β*_*w*2_: *k* = −0.8398 ± 0.1613, *P* = 2.1741 × 10^−7^; Fig. 6B and C). In addition, we found beta ERD was also significantly reduced during DBS condition compared with no DBS (CDBS versus no DBS: *k* = −0.8642 ± 0.2082, *P* = 3.5655 × 10^−5^; ADBS versus no DBS: *k* = −0.2439 ± 0.0990, *P* = 0.0138). However, the difference between CDBS and ADBS was not statistically significant (*k* = 0.3476 ± 0.1932, *P* = 0.0723). The results in the previous section showed that reaction time was more related to beta ERD, and the results presented here may explain why CDBS and ADBS lead to similar changes in reaction time.

Similarly, beta (ADBS: *k* = −0.2756 ± 0.0749, *P* = 0.0002; CDBS: *k* = −1.5111 ± 0.1910, *P* = 6.2992 × 10^−15^; Fig. 6E) and gamma (ADBS: *k* = −0.3937 ± 0.0767, *P* = 3.2493 × 10^−7^; CDBS: *k* = −2.6497 ± 0.2023, *P* = 1.8303 × 10^−36^; Fig. 6F) power during movement were significantly suppressed by DBS, and the suppression was stronger during CDBS compared with ADBS (Beta: *k* = −0.9337 ± 0.1189, *P* = 7.6942 × 10^−15^; Gamma: *k* = −1.8405 ± 0.1074, *P* = 2.3989 × 10^−60^; Fig. 6E and F). These results may explain why CDBS and ADBS lead to similar changes in movement speed: even though CDBS suppressed beta more than ADBS, it also suppressed gamma more, whereas both reduction of beta and increase of gamma contributed to invigorated movements.

## Discussion

There were three main findings from this study. First, we showed that shortening the smoothing window to 200 ms made the ADBS more responsive. Further, it shortened the average duration of beta bursts by increasing the number of bursts shorter than 200 ms. However, this did not bring any behavioural benefit compared to ADBS with a 400-ms smoothing window for estimating beta, supporting the argument that only long STN beta bursts are pathological in PD. Second, we showed that, although beta-triggered ADBS reduced the average time on stimulation during reaching movements, it did not compromise motor performance in terms of reaction time and movement speed compared with CDBS. Both ADBS and CDBS improved the performance of reaching and finger-tapping movements to a similar extent compared with no DBS. Third, our results indicated that, although ADBS achieved similar effect as CDBS in reducing bradykinesia and improving reaction time and movement speed, it was not as effective as CDBS in suppressing resting tremor.

### Why was there no behavioural difference between ADBS-200 and ADBS-400?

Previous studies have shown that STN beta bursts of different durations might have different roles in PD. In particular, the occurrence of longer beta bursts with large amplitude positively correlates with motor impairment,^10–13,39^ which has also been confirmed in animal models of PD.^40^ Here, in addition to the commonly used 400-ms smoothing time window (ADBS-400),^10,14–16^ we tested a faster ADBS algorithm, in which a 200-ms smoothing time window was used (ADBS-200), to test whether this might further improve the efficacy of ADBS. Our results showed no difference between these two ADBS conditions in any of the evaluated motor performance metrics, including reaction time, movement velocity, resting tremor, root-mean-square acceleration and blinded video ratings of finger-tapping (Fig. 3). Please note that here the blinded video ratings were conducted by two movement disorder specialists under the guidance of MDS-UPDRS-III (finger tapping instruction), which we believe is somewhat representative of the clinical assessment of bradykinesia. As shown in Fig. 3 (group level) and in Supplementary Fig. 2 (individual level), blinded video ratings did not differ between ADBS-200 and ADBS-400 but improved significantly during ADBS compared with no DBS. These results were unlikely due to errors in implementation of these two algorithms, as a *post hoc* analysis confirmed that ADBS-200 was more responsive to the beta oscillations leading to more frequent switching on/off of the stimulator (Fig. 2A), despite a similar total stimulation on time (Fig. 2B) compared with ADBS-400. We further compared how the two ADBS strategies modulated beta burst characteristics and found that ADBS-200 reduced the average beta burst duration compared with ADBS-400 (Fig. 2C) by increasing the number of shorter bursts with durations less than 200 ms, while keeping a similar number of longer bursts (Fig. 2D). These results further support the hypothesis that only long beta bursts (>400 ms) have a pathological effect in PD.^10–13^ Therefore, being more responsive to those short bursts with durations less than 400 ms appears unnecessary.

### Why did ADBS provide comparable improvement in motor performance to CDBS?

STN beta-triggered adaptive DBS has been shown to be at least as effective as conventional continuous DBS as evaluated by MDS-UPDRS-III in multiple studies,^8,14–21^ but it is still unclear whether beta-triggered ADBS is as effective when patients are engaged in a motor task, since STN beta is suppressed during movement initiation and execution.^22–24^ A recent study of three people with PD showed that ADBS might negatively affect the returning part of a reaching movement and delay movement termination,^17^ although motor improvement as measured by MDS-UPDRS-III was comparable to CDBS. In this study, we found that ADBS achieved similar effects as CDBS in improving motor performance in a reaching task in terms of reaction time, movement velocity, and in improving bradykinesia measured by root-mean-square acceleration and blinded video ratings of finger-tapping movements (Fig. 4). Therefore ADBS, despite reduced stimulation during ballistic reaching movements (Fig. 5C and D), did not appear to compromise movement initiation or execution compared with CDBS.

There are two explanations for this finding. First, even though beta power is reduced during movements when averaged across trials, transient episodes of long beta bursts can still be observed in individual trials.^41^ This explains why in this study, some stimulation (~30% of the time) was still delivered during movement under ADBS conditions (Fig. 5D and H). We hypothesize that long pathological beta bursts can still occur during movements, which can be curtailed by ADBS, leading to improvement in motor performance. Second, our analysis revealed that during reaching movement, the reaction time was not predicted by beta power *per se* but was predicted by beta ERD (Table 2), which was significantly reduced during DBS compared to no DBS with no difference between CDBS and ADBS. Previous studies have suggested that beta ERD represents cortical activation, while beta event-related synchronization (ERS) represents an inactive, idling state with reduced excitability of the cortex.^42^ Chen *et al*.^43^ found that during self-paced movements, corticospinal excitability increases and reaches a maximal level during movement initiation, then reduces after movement initiation, which is a very similar pattern to beta ERD during movement initiation. In a separate study from the same group,^44^ a negative correlation was found between single-trial STN beta power and corticospinal excitability during successful stopping movement in patients with PD. Thus, quicker movement initiation could be associated with a quicker de-activation of the corticospinal excitability as well as a quicker completion of beta desynchronization, resulting in a smaller STN beta ERD. On the other hand, a positive correlation was reported between the latency of STN beta ERD and reaction time in patients with PD using a go/nogo task, with shorter reaction times associated with earlier ERD onsets.^22,45^ Here we quantified beta ERD as the difference in beta power between two fixed time windows relative to movement initiation, thus, a smaller ERD could be due to an earlier ERD onset. However, these are still speculations; further exploration on this would require new data and is outside the scope of this work. Furthermore, our results revealed that reduced beta power and increased gamma power during movement together predicted faster movement speed (Table 2). Previous studies showed that gamma power in the human basal ganglia is positively correlated with movement speed in patients with either PD or dystonia.^46–49^ Here we show that both STN beta and STN gamma power during movement help predict movement speed, with significant negative and positive estimation effects for beta and gamma, respectively. However, both beta and gamma power were more strongly suppressed during CDBS compared with ADBS (Fig. 6). To better investigate the stimulation induced beta and gamma suppression on individual hemispheres, we further compared the resting (5-s before the Go-cue) beta and gamma power between no DBS and CDBS conditions. As shown in Supplementary Fig. 3, the suppression of beta (in 77.78% of recorded hemispheres) and gamma (in 83.33% of recorded hemispheres) power was consistent for most of the tested hemispheres. The stimulation induced power suppression in beta and gamma frequency bands shared similar spatial distributions relative to the STN, and positively correlated with each other (*r* = 0.8073, *P* = 5.1252 × 10^−5^, Pearson correlation). This suggests that although beta was better suppressed during CDBS, ADBS preserved gamma better which help invigorate movements, so that the overall movement speeds were similar during CDBS and ADBS conditions.

### Why was ADBS not as effective as CDBS in suppressing resting tremor?

Previous studies have demonstrated that STN beta oscillations positively correlate with the severity of bradykinesia and rigidity but not with resting tremor.^4–8,32,50–52^ Several existing trials testing the performance of STN beta-triggered ADBS in chronically implanted patients showed re-emergence of tremor during ADBS in some tremor-dominant people with PD, although its effectiveness with bradykinetic phenotypes has been demonstrated consistently.^18,53^ Indeed, a decrease of beta activity during parkinsonian tremor has been reported in several studies.^54,55^ In the presence of tremor, neuronal oscillations at tremor frequency (3–7 Hz) tend to increase in the cortical-basal ganglia-thalamic circuit,^56^ whereas beta power (13–30 Hz) and beta band coupling in the motor network are reduced.^54^ Our previous study also showed that in people with PD with pre-existing symptoms of tremor, successful volitional beta suppression through neurofeedback training was associated with an amplification of tremor, which correlated with increased theta band activity in STN LFPs.^35^ These results suggest that the underlying pathophysiology for tremor is different from that for bradykinesia and rigidity in PD. Both CDBS and ADBS significantly improved motor performance and resting tremor compared with no DBS. However, resting tremor was better suppressed during CDBS than ADBS (Fig. 4). These results suggest that apart from STN beta, an additional biomarker for resting tremor might be required while developing ADBS strategies for simultaneous control of bradykinesia/rigidity and tremor in PD.

### Remaining challenges for the development of ADBS systems for Parkinson’s disease

The results of this study have implications for the further development of ADBS systems for PD. First, we confirmed that tracking the fast beta dynamics using a short smoothing time window does not bring any additional advantage compared to the 400 ms windows used in previous trials. This may inform future studies on the design of more sophisticated controllers (e.g. proportional-integral-derivative, PID), in which the temporal dynamics of the beta oscillations are taken into account, and the interactions between the controller and the targeted brain oscillations will be more complicated. On the other hand, more research effort should be invested in addressing the remaining issues of stimulation artefacts and self-triggering related to the fast termination of stimulus trains.^31^ In our study, a 250-ms ramping up/down during each switching on/off plus a 50-ms refractory time after each switching off were used to minimize this issue. However, this could be improved at a hardware level.^57^ Alternatively, continuous modulation of the stimulation intensity using proportional control could also remove the self-triggering problem. Second, it might be more beneficial to combine STN beta, gamma, and real-time detection of the patient’s movement status in creating an enhanced adaptive stimulation algorithm. Several previous studies have demonstrated the feasibility of detecting movement state based on bioelectrical signals recorded from the cortical-basal ganglia-thalamic circuit in people with PD or essential tremor.^21,58–61^ Suppressing beta while minimizing the suppression of gamma during movement might result in improved motor performance. However, extracting gamma power in real-time using currently available chronically implanted devices is very challenging for several reasons including: (i) stimulation artefact, which has a bigger impact on gamma than beta since it is closer to the stimulation frequency; (ii) lower signal-to-noise ratio in the gamma band, since gamma activity has a smaller amplitude than beta; and (iii) a higher sampling rate, larger cutting frequency of the anti-aliasing filtering and higher resolution of the analogue to digital conversion (ADC) are required to record physiological gamma band activities. Despite this, with the currently available implantable, miniaturized systems such as the Activa PC+S (Medtronic), it has been possible to ‘sense’ cortical gamma band activities which have been related to treatment-induced dyskinesia.^62^ The Summit RC+S (Medtronic) has also been used to track two biomarkers simultaneously, i.e. subcortical beta and cortical gamma, to distinguish mobile and immobile states for ADBS,^21^ or gamma and theta-alpha oscillations for independent PD and sleep state detection, respectively.^63^ Recently, Vaou *et al*.^64^ used Percept (Medtronic) to monitor STN beta and gamma oscillations for akinetic-rigid and dyskinetic symptoms, respectively, in patients with PD. Therefore, although some of the functions are not implemented in the currently existing commercialized device, it should still be possible to estimate beta and gamma at the same time and utilize both biomarkers for ADBS in implantable devices. Alternatively, STN DBS at a lower frequency than the standard 130 Hz (e.g. 60 Hz) may be a workaround, as it has been suggested to be of benefit for axial features (freezing of gait, postural instability, speech, swallowing function, etc.) in patients with PD.^65–68^ This could potentially be due to better preservation of gamma, whilst suppressing beta with lower stimulation frequency, although this is yet to be established. In addition, when gamma oscillation is to be used as a feedback signal, movement-related gamma increase, which tends to correlate with movement speed, needs to be differentiated from finely-tuned gamma, which might be an indicator of dyskinesia.^69^ Third, additional feedback signal(s) apart from STN beta might be required to develop an ADBS system for tremor-dominant people with PD. Although ADBS does not necessarily mean less energy consumption by the implantable pulse generator (IPG), since in general less energy will be delivered to the brain, it may still be beneficial in reducing stimulation-induced side effects. With improved strategies for applying ADBS, it is possible that ADBS will provide better clinical improvement than CDBS for people with PD in the future.

### Limitations

All experiments for this study were conducted 3–6 days after the first surgery for DBS electrode implantation, when the post-operative stun effect was appreciable. In addition, the stimulation configurations used in this study, such as ring-mode construction for directional DBS leads, selection of the stimulation contact, amplitude etc., could be suboptimal and different from those used in clinical practice. Therefore, the effect of DBS in general could be further improved. However, the same stimulation parameters were used in all tested DBS conditions within each patient, allowing for a fair comparison between the different conditions. Although we did not see significant difference in any of the assessed discrete upper limb fine motor tasks between ADBS-200 and ADBS-400, other parkinsonian symptoms, such as rigidity, balance and other axial functions were not assessed in this study. Therefore, the effects of beta triggered ADBS using different smoothing windows on those parkinsonian symptoms require further investigation. Here, only a relatively simple form of ADBS based on thresholding and the effects of different smoothing windows was tested. It would be interesting to test the effects of varying other aspects such as different thresholds and/or using a more sophisticated controller such as PID for continuous modification of different stimulation parameters beyond stimulation intensity for ADBS. However, regardless of the control algorithms used, the smoothing window for quantifying the beta amplitude as the feedback signal is a key parameter that needs to be considered. The modelling results in this study showed that reaction time was predicted by STN beta ERD, while mean velocity during reaching movement was predicted by STN beta and gamma power, but whether the relationships are causal remains unanswered. Another limitation is that only shortterm effects of DBS were considered during two specific motor tasks, i.e. ballistic reaching and finger-tapping. It is unclear to what degree the achieved results could be generalized to longer experimental periods, especially when patients are engaging in normal activities of daily living.

## Conclusion

This study evaluated the effectiveness of STN beta-triggered ADBS during a reaching task involving upper-limb movements in 13 people with PD. We showed that beta-triggered ADBS did not compromise the motor performance of cued reaching movements in terms of reaction time and movement speed compared with CDBS. ADBS and CDBS significantly improved motor performance by similar amounts compared with no DBS. In addition, we demonstrated that using a shorter smoothing window to estimate beta did make ADBS more responsive. It shortened beta burst durations by increasing the number of beta bursts shorter than 200 ms, but this did not bring any additional benefit in motor performance. We also showed that both STN beta reduction and gamma power increase during movement helped in predicting movement speed, suggesting that combining beta, gamma and movement status might confer added benefit in ADBS. In addition, beta-triggered ADBS was not as effective as CDBS in suppressing parkinsonian resting tremor, suggesting that additional feedback signals might be required for tremor-dominant patients. These findings have significant implications for the further development of ADBS algorithms to improve the treatment for PD.

## Supplementary Material

Supplementary materials and methods

Supplementary video 1

Supplementary video 2

## Figures and Tables

**Table 1:** Details of the stimulation used during the recording of this study and in clinical settings

Case	DBS lead	Experimental DBS				Chronic DBS	
		Stim contact (L/R)	Stim Amp (L/R, mA)	Bipolar feedback channel (L/R)	Online filter range (L/R Hz)	Stim Contact (L/R)	Stim Amp (L/R)
1	Medt1	L3^a^	3	L24	19–25	L2	3.3 V
2	Medt1	L3^b^/R2^c^	3.5/1.5	L24/R13	14–20/15–21	L1/R2	2.9/2.7 mA
3	Bost1	L2^c^/R3^c^	3/2	L13/R24	15–21/14–20	L2-L3/R2-R3	4.0/3.5 mA
4	Bost2	L3^c^	1	L24	16–22	L2-L3	4.2 mA
5	Abbo	R3^a^,^b^	1.5	R24	17–23	R2	3.2 mA
6	Medt2	R2^a^	1.5	R13	19–25	R1	2.6 mA
7	Bost3	L2^c^/R2^c^	2.5/2.5	L13/R13	16–22/22–28	L2-L4/R2	2.8/2.3 mA
8	Medt2	R2^c^	3	R13	15–21	R2	3.6 mA
9	Medt2	L3^a^	1.5	L24	14–20	L4	2.5 mA
10	Medt2	L2^c^/R2^c^	1/3	L13/R13	22–28/22–28	L2/R2	2.4/3.5 mA
11	Medt2	L3^a^/R2^c^	3.5/3.5	L24/R13	18–24/17–23	L2/R2	1.9/1.7 mA
12	Medt2	L2^c^/R2^c^	3/3	L13/R13	12–18/21–27	L2/R2	1.0/1.0 mA
13	Bost1	L2^c^/R2^c^	2/2	L13/R13	18–24/20–26	L2-L3/R2-R3	4.5/1.7 mA
Mean	–	–	2.38	–	17.3–23.3	–	2.77
SEM	–	–	0.18	–	0.66	–	0.22

Abbo = St. Jude Medical Infinity 0.5 mm spaced directional DBS leads with 1-3-3-1 configuration, Abbott; Amp = amplitude; Bost1 = Vercise™ directional lead with 1-3-3-1 configuration, Boston Scientific; Bost2 = Cartesia™ X leads with 3-3-3-3-3-1 configuration, Boston Scientific; Bost3 = Cartesia™ HX leads with 3-3-3-3-1-1-1-1 configuration, Boston Scientific; DBS = deep brain stimulation; L = left; Medt1 = Quadripolar non-directional Macroelectrode, Model 3389, Medtronic; Medt2 = SenSight™ 0.5 mm spaced directional lead with 1-3-3-1 configuration, Medtronic; R = right; SEM = standard error of the mean; Stim = stimulation.

a The contacts in experimental and chronic DBS appeared at adjacent levels.

b Hemispheres excluded from analysis (see text for detailed reasons).

c The contacts in experimental and chronic DBS appeared at the same level.

**Table 2:** Effects of beta/gamma power in predicting motor performance during reaching movement revealed by generalized linear mixed effect modelling

**Predicting reaction time**									
**Model 1: RT *~* 1 + *k*_1_condID × *k*_2_*β*_*w*1_ + 1|HemID**									
**AIC**	***k*_1_**	***p*_1_**	***k*_2_**	***p*_2_**	***k*_inter_**	***p*_inter_**	–	–	***R^2^***
1438.9	0.0349 ± 0.1337	0.7943	0.0097 ± 0.0087	0.2616	−0.0049 ± 0.0048	0.3128	–	–	0.2456
**Model 2: RT ~ 1 + *k*_1_condID × *k*_2_*β*_*w*2_ + 1|HemID**									
**AIC**	***k*_1_**	***p*_1_**	***k*_2_**	***p*_2_**	***k*_inter_**	***p*_inter_**	–	–	***R* ^2^**
1434.7	−0.0238 ± 0.1385	0.8637	−0.0054 ± 0.0099	0.5877	−0.0035 ± 0.0054	0.5166	–	–	0.2462
**Model 3: RT ~ 1 + *k*_1_condID × *k*_2_*β*_erd_ + 1|HemID**									
**AIC**	***k*_1_**	***p*_1_**	***k*_2_**	***p*_2_**	***k*_inter_**	***p*_inter_**	–	–	***R* ^2^**
1432.9	−0.0742 ± 0.0363	**0.0409**	0.0301 ± 0.0150	**0.0453**	−0.0118 ± 0.0085	0.1636	–	–	0.2478
**Compare (Model 1, Model 3)**				**Compare (Model 2, Model 3)**					
**LRStat**	**deltaDF**		***P***	**LRStat**		**deltaDF**			***P***
6.0748	0		**<0.001**	1.8418		0			**0 < 0.001**
**Predicting MV**									
**Model 4: MV ~ 1 + *k*_1_condID + *k*_2_rrID + *k*_3_*β*_mov_ + 1|HemID**									
AIC	***k*_1_**	***p*_1_**	***k*_2_**	***p*_2_**	***k*_3_**	***p*_3_**	–	–	***R*_2_**
−6481.9	0.0112 ± 0.0039	**0.0041**	−0.0710 ± 0.0034	**<0.001**	−0.0006 ± 0.0006	0.3147	–	–	0.6746
**Model 5: MV ~ 1 + *k*_1_condID + *k*_2_rrID + *k*_3_γ_mov_ + 1|HemID**									
**AIC**	***k*_1_**	***p*_1_**	***k*_2_**	***p*_2_**	***k*_3_**	***p*_3_**	–	–	***R* ^2^**
−6490.6	0.0139 ± 0.0039	**0.0004**	−0.0714 ± 0.0034	**<0.001**	0.0018 ± 0.0006	**0.0019**	–	–	0.6752
**Model 6: MV ~ 1 + *k*_1_condID + *k*_2_rrID + *k*_3_*β*_mov_ + *k*_4_*γ*_mov_ + 1|HemID**									
**AIC**	***k*_1_**	***p*_1_**	***k*_2_**	***p*_2_**	***k*_3_**	***p*_3_**	***k*_4_**	***p*_4_**	***R* ^2^**
−6513.3	0.0142 ± 0.0039	**0.0003**	−0.0689 ± 0.0034	**<0.001**	−0.0042 ± 0.0008	**6.7338 × 10^−7^**	0.0049 ± 0.0009	**7.7075 × 10^−9^**	0.6773
**Compare (Model 4, Model 6)**				**Compare (Model 5, Model 6)**					
**LRStat**	**deltaDF**		***P***	**LRStat**		**deltaDF**			***P***
33.35	1		**7.6987 × 10^−9^**	24.685		**1**			**6.7514 × 10^−7^**

*β*_*w*1_ = average beta power during 1 to 0.5 s before movement initiation (W1 in Fig. 6A); *β*_*w*2_ = average beta power during 0.2 s around movement initiation (W2 in Fig. 6A); *β_erd_* = *β*_*w*1_ − *β*_*w*2_; *β_mov_* = average beta power during movement (from reach/return movement onset to target reached); *γ*_mov_ = average gamma power during movement. Models 1–3 considered reach movements only, since reaction time and *β_erd_* were quantified only for reach movements. Models 4–6 considered all reach and return movements. AIC = Akaike information criterion; condID = stimulation condition index; deltaDF = difference in degrees of freedom between two models; HemID = hemisphere index; inter = interaction; LRStat = likelihood ratio test statistic for comparing two models; MV = mean velocity; rrID = reach or return index; RT = reaction time.

## Data Availability

The data and codes will be shared on the data sharing platform of the MRC Brain Network Dynamics Unit: https://data.mrc.ox.ac.uk/mrcbndu/data-sets/search.
