# Supplementary material for: Beta-triggered adaptive deep brain stimulation during reaching movement in Parkinson’s disease
Source: Brain. Author manuscript; Available in PMC 2023 Dec 2. (PMC10690014; doi:10.1093/brain/awad233)
Supplement: Supplementary materials and methods [file EMS191520-supplement-Supplementary_materials_and_methods.pdf]

Supplementary materials and methods

Stimulation

The implanted DBS leads were Medtronic non-directional 3389 (2 cases), Medtronic SenSight™ directional with configuration 1-3-3-1 (6 cases), Boston Vercise™ directional with configuration 1-3-3-1 (2 cases), Boston Cartesia X directional with configuration 3-3-3-3-3-1 (1 cases), Boston Cartesia HX directional with configuration 3-3-3-3-1-1-1-1 (1 cases), or Abbott Infinity™ directional with configuration 1-3-3-1 (1 case). For consistency, in cases with directional leads, the segmented contacts were used in ring mode. In cases with Boston Cartesia X/HX directional leads, only the most inferior 4 levels which were supposed to locate in STN were considered for analysis.

Selecting stimulation contact and amplitude, and the beta frequency band for feedback

Specifically, we delivered continuous DBS to one of the middle two contacts initially at 0.5 mA. We then progressively increased the amplitude by 0.5 mA increments, until clinical benefit was seen without side effects such as paraesthesia, or until 3.5 mA was reached as the maximum amplitude. If no apparent clinical effect was observed, we repeated this procedure for the other middle contact level. Once the stimulation contact and amplitude were selected, a period of 2 minutes of rest recordings were performed. LFPs were recorded from two contacts neighbouring the selected stimulating contact in the differential bipolar mode. To select the individualized beta frequency band for feedback, the recorded LFPs were first notch-filtered at 50 Hz and band-pass filtered between 1 and 95 Hz using a second order zero-phase digital filter. The periodogram power spectral density (PSD) was then estimated. The feedback beta frequency band was selected as ± 3 Hz around the largest beta peak (13-30 Hz).

Kinematic data analysis

Finger-tapping: The root-mean-square acceleration was quantified for each individual block and then normalized against the maximum across all blocks within the same hand. In addition, two experienced movement disorder specialists (F.B. and A.M. in the author list) reviewed the recorded videos of the finger-tapping movements and separately rated the movement according to adjusted MDS-UPDRS-III (finger tapping instruction). The assessors were blinded to the

stimulation condition of each video. The mean rating from the two assessors was then calculated. Due to obvious fatigue effect after long-lasting finger-tapping, only the first 10 s accelerometer measurements and video recordings were considered during the assessment.

Resting tremor: To investigate the impact of beta-triggered ADBS on resting tremor, we quantified tremor power using the accelerometer measurements recorded from the tested hand 5 s before the Go-cue, when the patient was at rest. More specifically, the recorded three-axes accelerometer signals were first band-pass filtered between 1 and 95 Hz and band-stop filtered between 48 and 52 Hz using 3-order zero-phase Butterworth filters, then decomposed into time-frequency domain using continuous Morlet wavelet transformation with 6 cycles, and a linear frequency scale ranging from 1 to 10 Hz at 0.5 Hz resolution. The average power in tremor frequency band (i.e., 3-7 Hz) across three axes were quantified (10log10 transferred to dB) as the resting tremor severity.

Stimulation and LFP data analysis

To investigate the movement related modulation in the STN, the epoched LFPs were band-pass filtered between 1 and 95 Hz, band-stop filtered between 48 and 52 Hz using 3-order zero-phase Butterworth filters, and decomposed into time-frequency domain using continuous Morlet wavelet transformation with a linear frequency scale ranging from 1 to 95 Hz at 1 Hz resolution, and a linearly spaced number (4–8) of cycles across all calculated frequencies. The calculated power of each time point at each frequency was decibel (dB) baseline normalized against the average power in the 1 second window before the Go-cue. The beta and gamma power were also quantified as the average power in the frequency bands of 13-30 Hz and 35-90 Hz, respectively.

Supplementary Table 1 Clinical details of all recorded participants

Case	G	Age (yr)	DD (yr)	H	UPDRS (OFF/ON)	Centre	Pre-dominant symptom(s) before surgery	Drugs (total daily dose)	LEDD reduction with DBS (%)
1	M	61-65	13	R	30/9	KCH	Motor fluctuations, dyskinesia, unpredictable OFF periods	Half Sinemet CR 25mg/100mg OD, Opicapone 50mg OD, Rasagiline 1mg OD, Ropinirole 20mg OD, Sinemet 125mg × 5/day	42.20
2	M	71-75	8	R	51/8.5	KCH	Tremor, gait	Sinemet Plus (25mg/100mg) 2 tablets QDS, Sinemet CR (50mg/100mg) OD, Rasagiline 1mg OD, Ropinirole XL 4mg OD, Amantadine 100mg OD	-27.96*
3	M	56-60	11	R	41/16	SGH	Bradykinesia, dyskinesia	Sinemet 25/100 and 50/12.5, 5/day, rasagaline 1mg, opicapone 50mg	65
4	M	56-60	6	L	31/4	SGH	Tremor	Stanek 125/31.25/200, 4/day, propranolol 40mg 3/day, co-careldopa 100/25 MR 2/day, safinamide 100mg, Amantadine 100mg 1/day, clonazepam 500mcg 3 tablets at night, melatonin 2mg at night	70
5	F	46-50	6	R	53.5/20	KCH	Rigidity, dyskinesia	Apomorphine 0.9 ml per hour flow rate, Sinemet CR 250mg OD, Entacapone 200MG OD	72.79
6	F	61-65	10	R	29/6	SGH	Dyskinesia	Sinemet 25/100 dispersible once in the morning, sinemet 25/100mg 4/day, opicapone 50mg, rasagiline 1mg, sinemet MR 25/100 once at night	27
7	M	61-65	20	R	51/27	SGH	Tremor	Rasagiline 1mg, Ropinirole XL 4mg once a day, ropinirole 2mg twice a day, madopar 25/100mg and 12.5/50mg taken 4/day	28
8	F	66-70	6	R	43/13	KCH	Tremor, bradykinesia	Sinemet Plus (25MG/100MG) QDS	0&
9	F	61-65	15	R	26/13	SGH	Tremor, dyskinesia	Rasagiline 1mg, Stalevo 100/25/200 taken 4/day, amantadine 100mg once a day	31
10	F	66-70	6	R	16/6	SGH	Tremor	Sinemet 25/100 taken 4/day, opicapone 50mg	25
11	M	56-60	15	R	42/11	KCH	Tremor	Amantadine 100mg BD, Madopar 125mg QDS, Rasagiline 1mg OD	85.71
12	M	66-70	6	L	33/13	KCH	Tremor	Madopar 150 mg every 3 hours, Madopar CR (100MG/25MG) OD, Sertraline 100mg OD, Propranolol 10 mg TDS, Clonazepam 1mg OD	46.15
13	F	56-60	8	R	35/15	SGH	Rigidity, gait	Rasagiline 1mg, Ropinirole MR 10mg, Sinemet 25/100, 5/day	10
Mean		62.15	10		37.04/12.42				45.71
SEM		1.58	1.21		2.95/1.67				20.20

G=gender; yr=year; DD=disease duration; H=handedness; UPDRS=Movement Disorders Society Unified Parkinson's Disease Rating Scale part III; OFF/ON=OFF/ON medication; M=male; F=female; R=right; L=left; KCH=King's College Hospital; SGH=St George's Hospital; SEM=standard error of the mean; LEDD=Levodopa equivalent daily dose. *This patient was anxious and claimed to have developed new symptoms while reducing LEDD. &This patient had very low LEDD pre surgery (400 g). These two patients did not include in the calculation of Mean and SEM of the LEDD reduction with DBS.

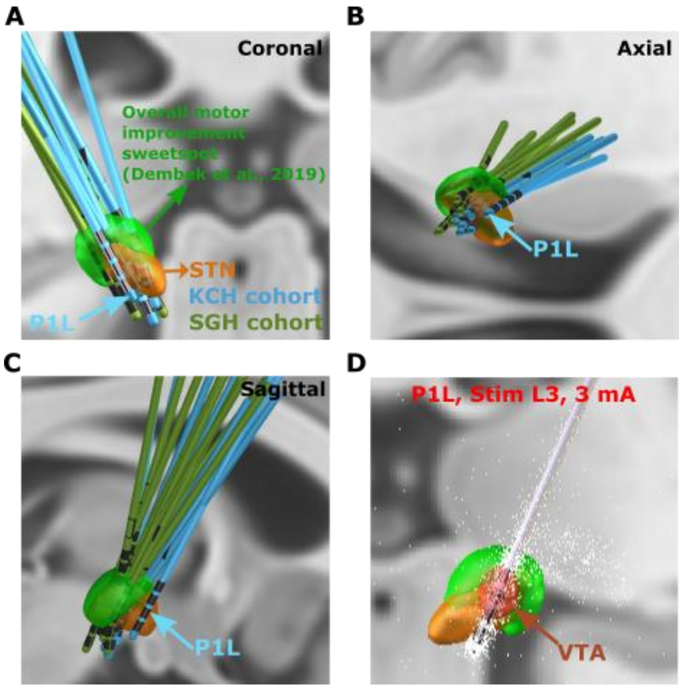

Supplementary Figure 1. Volume-of-tissue activated (VTA) analysis on electrode P1L. (A)-(C) Three-dimensional reconstruction in coronal (A), axial (B), and sagittal (C) views of all analysed DBS leads localized in standard MNI-152_2009b space using Lead-DBS, the same as **Fig. 1D**. **(D)** Simulated VTA for electrode P1L when applying 3 mA stimulation at channel 3 using Lead-DBS. Please note that this electrode was targeted in the left hemisphere, but was mirrored and shown in the right hemisphere in **Fig. 1D** and here in plots A-C.

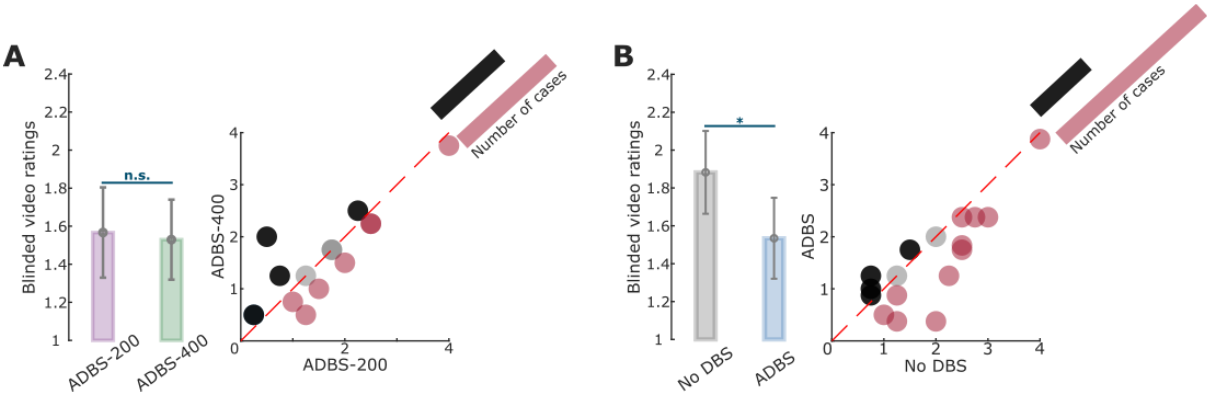

Supplementary Figure 2. Comparison on blinded video ratings between ADBS-200 and ADBS-400 (A), and between ADBS and no DBS (B). The error bar plots on the left show the mean and SEM across all tested hemispheres in different conditions; *p<0.05; n.s.: not significant; p-values were quantified using generalized linear mixed effect modelling on block basis. The dots indicate the means for each tested hemisphere. The black- and red-shaded dots indicate higher measurement in ADBS-400 and ADBS-200 conditions (A) respectively, or in ADBS and no DBS conditions (B) respectively. The gray-shaded dots indicate no difference between two conditions. The bar on the diagonal refers to the number of cases with higher measurement in each condition.

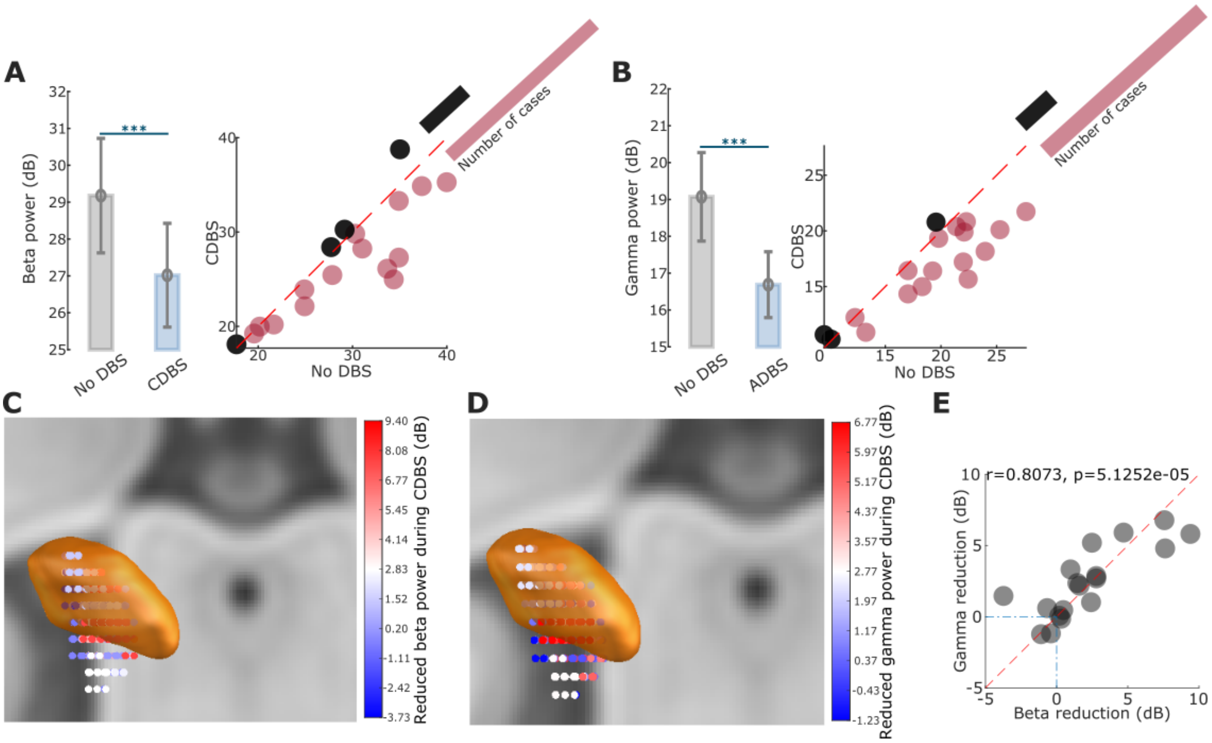

Supplementary Figure 3. Resting beta and gamma power were suppressed with continuous DBS for most of the tested hemispheres, and stimulation induced reduction of beta and gamma shared similar distributions relative to STN, and positively correlated with each other. (A)-(B) Comparison of resting beta (A) and gamma (B) power between no DBS (x-axis) and CDBS (y-axis). The error bar plots on the left show the mean and SEM across all tested hemispheres in different conditions; ****p*<0.001; *p*-values were quantified using generalized linear mixed effect modelling on trial basis. The dots indicate the means for each tested hemisphere. The black- and red-shaded dots indicate higher measurement in CDBS and no DBS conditions, respectively. The bar on the diagonal refers to the number of cases with higher measurement in each condition. **(C)-(D)** Distributions of stimulation induced beta (C) and gamma (D) reduction relative to STN in the standard MNI space. **(E)** Correlation between beta (x-axis) and gamma (y-axis) reduction with CDBS.

Supplementary Video 1: Reaching task (P4 right hand)

Supplementary Video 2: Finger-tapping task (P11 right hand)
